# *Salmonella* Infantis in Broiler Flocks in Slovenia: The Prevalence of Multidrug Resistant Strains with High Genetic Homogeneity and Low Biofilm-Forming Ability

**DOI:** 10.1155/2019/4981463

**Published:** 2019-02-07

**Authors:** Mateja Pate, Jasna Mičunovič, Majda Golob, Lene Karine Vestby, Matjaž Ocepek

**Affiliations:** ^1^University of Ljubljana, Veterinary Faculty, 1000 Ljubljana, Slovenia; ^2^Norwegian Veterinary Institute, 0106 Oslo, Norway

## Abstract

For almost a decade, the number of* Salmonella enterica* subspecies* enterica* serovar Infantis-positive broiler flocks has been steadily increasing in Slovenia, doubling the number of positive holdings in only a few years. Since multidrug resistant* S*. Infantis isolates are highly prevalent in the broiler meat industry and may represent a public health concern through the food chain, we aimed to investigate the antimicrobial susceptibility, genetic diversity, and biofilm-forming ability of* S*. Infantis from Slovenian broiler flocks. A total of 87* S*. Infantis strains isolated from broiler faeces in the period between 2007 and 2013 were studied. The samples originated from 41 farms which were subcontractors of three major food business operators and from two autonomously operating holdings (farms). Isolates were phenotypically tested for their susceptibility to 14 antimicrobials from nine classes by determining the minimum inhibitory concentration with the microdilution method. Only 8% of the isolates were susceptible to all of the antimicrobial agents tested, while 88.5% of the isolates were multidrug resistant, with the most common resistance pattern CipNxSSuT (65.5%) followed by CipNxSuT (17.2%). Pulsed-field gel electrophoresis (PFGE) divided the strains into five clusters (A-E) comprising 16 distinct* Xba*I PFGE profiles. Sixty-five out of 87 isolates were grouped in clusters A and B, with the predominant PFGE profiles A1 and B1 encompassing 33 and 28 isolates, respectively. A vast majority of the isolates (75/87) showed >90% PFGE profile similarity. The biofilm-forming capacity of the tested isolates, determined with crystal violet assay in polystyrene microwell plates, was generally weak. The average biofilm formation for persistent strains was higher than for presumably nonpersistent strains; however, the difference was not significant. It seems that* S*. Infantis persistence on broiler farms is more related to its widespread occurrence in the broiler production chain and ineffective disinfection protocols than to its ability to form biofilm.

## 1. Introduction

The presence of* Salmonella* in poultry is considered to be a risk factor for the contamination of meat and eggs. In order to prevent zoonotic transmission of* Salmonella*, national control programmes for* Salmonella* in poultry are set in the European Union (EU) to reduce the prevalence of certain serovars [1]. Most of the EU Member States met their* Salmonella* reduction targets for poultry, and* Salmonella* prevalence is declining in these animal populations. At the same time, a significant declining trend of human salmonellosis cases was observed between 2008 and 2012 [2]. However,* Salmonella* still ranks first among the causative agents of food-borne outbreaks in the EU. The two most commonly reported* Salmonella *serovars in humans in 2017 were* S. *Enteritidis and* S. *Typhimurium [2].

A statistically significant decreasing trend of confirmed human salmonellosis cases was observed between 2008 and 2017; however, during the last five years (2013-2017), the overall trend has not shown any statistically significant decrease or increase [2]. In contrast,* Salmonella enterica* subspecies* enterica* serovar Infantis (*S*. Infantis) became the emerging nontyphoidal* Salmonella* worldwide. In the EU,* S*. Infantis was the most commonly reported serovar from broiler flocks and broiler meat.* S*. Infantis is an important public health concern due to its frequent isolation from humans; it ranks in fourth position among top-10 human serovars [2]. It is frequently multidrug resistant (MDR) and seems successfully spread by certain clones among broilers and humans [3]. MDR* S*. Infantis isolates are highly prevalent in the broiler meat industry in several EU Member States and contribute significantly to the overall occurrence of MDR* Salmonella* in Europe [3]. Resistant strains may spread from animals to humans through the food chain, representing a public health concern. As shown before, the same MDR* S*. Infantis clone was recovered from the broiler houses, abattoirs, retail meat, and humans [4]. Furthermore, a recent study demonstrated the spread of ESBL-producing MDR* S*. Infantis from animals to humans in Italy [5].

Since 2010, a considerable increase of* S*. Infantis-positive broiler flocks has been observed in Slovenia. The number of positive flocks has increased by more than 100% in only two years. In addition,* S*. Infantis has been often repeatedly detected in certain holdings, despite applying sanitation measures during the production break [6]. It is well known that the elimination of* Salmonella* from poultry houses is a difficult task and that cleansing and disinfection methods may often be ineffective in a field situation where protective organic materials are abundant [7, 8]. Furthermore, it has been demonstrated that production of fimbriae and cellulose and the ability to form biofilm are important for the survival of* Salmonella* on surfaces and persistence in the environment [9, 10]. To date, only a few studies on biofilm-forming ability of* S*. Infantis from poultry have been published [8, 11, 12].

Because of an alarming spread of* S.* Infantis in the broiler chicken industry in recent years, the aim of this study was to get some insight into the genetic diversity and antimicrobial susceptibility of* S.* Infantis from broiler flocks. However, as the data obtained indicated the persistence of specific clones in certain farms, the biofilm-forming capacity of the isolates has also been tested, providing relevant data on a large collection of* S.* Infantis isolates.

## 2. Materials and Methods

### 2.1. Bacterial Strains and Culture Conditions

Eighty-seven* S*. Infantis strains isolated from broiler faeces in the period between 2007 and the first half of 2013 were included in this study (Table 1). The broiler chicken originated from at least 41 holdings (farms); the origin is not known for four isolates, hence the term ‘at least.' The holdings (farms) were subcontractors of three major food business operators (FBOs; SI-A, SI-B, and SI-C). In addition, chicken from two autonomously operating holdings (AOHs; SI-X) were investigated. In total, there were 17 subcontractors of FBO SI-A, at least two subcontractors of FBO SI-B, and 22 subcontractors of FBO SI-C. Seventeen holdings were sampled more than once in the time range from 14 days to four years (Table 1). The number of isolates collected at respective holdings was two to eight.

### 2.2. Antimicrobial Susceptibility Testing

**Figure 1:**
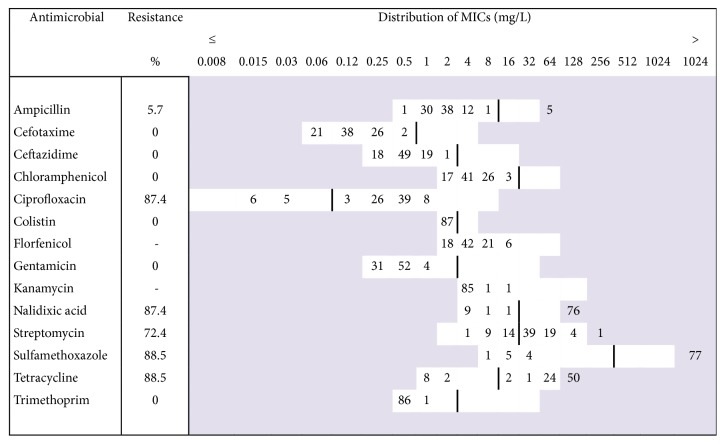
Distribution of MICs and resistance (%) in* Salmonella* Infantis isolates (n=87) from broilers, 2007–2013.

The antimicrobial susceptibility was evaluated by determining the minimum inhibitory concentration (MIC) by microdilution method using commercially available microplates (EUMVS2, Sensititre®, Trek Diagnostic Systems, Thermo Fisher Scientific, USA). All isolates were phenotypically tested for their susceptibility to 14 antimicrobials from nine different antimicrobial groups: ampicillin, cefotaxime, ceftazidime, chloramphenicol, ciprofloxacin, colistin, florfenicol, gentamicin, kanamycin, nalidixic acid, streptomycin, sulfamethoxazole, tetracycline, and trimethoprim.* Escherichia coli* ATCC 25922 was used as a test control strain. The results were interpreted according to the European Committee on Antibiotic Susceptibility Testing (EUCAST) epidemiological cut-offs [13] and the recommendations of the European Union Reference Laboratory for Antimicrobial Resistance [14]. The interpretative criteria were in concordance with the Decision 2013/652/EU of the European Commission [3]. Resistance to antimicrobials for which no breakpoint is available was shown as distribution of MICs (Figure 1). Multidrug resistance was defined as resistance to three or more antimicrobial classes.

### 2.3. Pulsed-Field Gel Electrophoresis (PFGE)

PFGE was carried out according to the standardised PulseNet protocol [15]. Restriction patterns were analysed with BioNumerics software (v. 6.6, Applied Maths, Belgium). The relation between two isolates was scored using the Dice coefficient of similarity. A cluster analysis was performed by the unweighted pair-group method with arithmetic means (UPGMA). Position tolerance and optimisation were set at 1%. Bands of size less than 33.3 kb were excluded from the analysis. Isolates with PFGE profiles of >95% similarity were considered to belong to the same cluster (marked by letters). Within a cluster, profiles differing from each other in at least one band were considered as subtypes (marked by digits).

### 2.4. Classification of Persistent and Presumably Nonpersistent Strains

If strains with the same PFGE profile had been isolated from the same poultry house over a time period of at least one year, the strain that was last isolated was classified as persistent. A strain was classified as presumably nonpersistent if no other strain with the same PFGE profile had been isolated from the same poultry house, or if the strains with the same PFGE profile had been isolated in a certain poultry house over a period of less than a year.

### 2.5. Biofilm-Forming Capacity Testing

All strains were stored at -80°C in brain heart infusion broth (BHI; Difco, BD, NJ, USA), supplemented with 15% glycerine (Merck KGaA, Darmstadt, Germany) and recovered on sheep blood agar at 37±1°C overnight. The bacterial cultures were then transferred into Luria Bertani broth (LB; Merck KGaA) and incubated statically overnight at 37±1°C. The assay was based on the method described by Vestby et al. [9]. In short, 20 *μ*l aliquots of overnight cultures were added to each well in a 96-well microtiter plate (Nunc Nunclon, Roskilde, Denmark) containing 180 *μ*l of LB without NaCl (LB ^wo^/NaCl; bacto-tryptone 10 g/l, yeast extract 5 g/l). Triplicates of each strain were used. After inoculation, the plates were incubated statically for 48±1 hour at 20±1°C. After incubation, optical densities (OD_595_) were measured before the plates were gently washed once with sterile distilled water (SDW). The plates were dried at room temperature before the addition of 220 *μ*l of 1% crystal violet (Sigma-Aldrich, St. Louis, MO, USA). After a 30-minute incubation at room temperature, the plates were washed three times with SDW. Then, 220 *μ*l ethanol:acetone (70:30, w:w) was added, followed by the incubation for 10 minutes at room temperature. OD_595_ were measured after the bound dye was dissolved using ethanol:acetone. For each strain, the result was calculated by subtracting the median OD_595_ of the three parallels of the control (test broth only) from the median OD_595_ of the three parallels of the sample. Two independent experiments were performed using freshly prepared reagents and media.

### 2.6. Statistics

Statistical analyses were performed using Excel® (Microsoft®, Redmond, WA, USA).

## 3. Results

### 3.1. Antimicrobial Susceptibility Testing

The majority of isolates (80/87; 92%) were resistant to at least one or more antimicrobials, and only 7 out of 87 isolates (mostly from year 2007) were susceptible to all 14 antimicrobials tested. Multidrug resistance was detected in 88.5% (77/87) of the isolates. By far the most common resistance pattern was CipNxSSuT, expressed by 65.5% (58/87) of the isolates. Resistance pattern CipNxSuT was the second most frequent, despite being exhibited by a considerably lesser number of isolates (15/87; 17.2%). The highest proportion of resistance was observed in tetracyclines (88.5% of isolates), sulfonamides (88.5%), and quinolones (nalidixic acid and ciprofloxacin, 87.4% each). The resistance to streptomycin was also notable (72.4%). Reduced susceptibility was found for ampicillin (5.7%). Acquired resistance to third-generation cephalosporins, chloramphenicol, colistin, gentamicin, and trimethoprim was not detected. The results indicate that the selected isolates did not belong to the group of extended-spectrum *β*-lactamase producing (ESBL) bacteria. Distribution of MICs is shown in Figure 1.

### 3.2. PFGE

**Figure 2:**
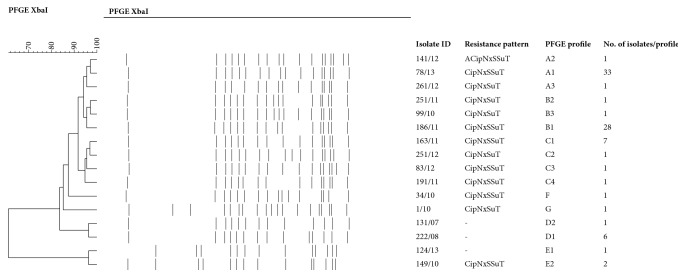
Sixteen representative PFGE profiles of* Salmonella* Infantis isolates selected from a total of 87 isolates included in the study.

A remarkable genetic homogeneity was noticed as a vast majority of isolates (75/87) showed >90% profile similarity. Five clusters (A to E) and 16 profiles (A1-3, B1-3, C1-4, D1-2, E1-2, F, and G) were defined. Representative PFGE profiles are shown in Figure 2.

Cluster A comprised 35 isolates originating from 12 subcontractors of FBO SI-A, 11 subcontractors of FBO SI-C, and both AOHs (Table 2). Almost all (94.3%) of the isolates in cluster A expressed PFGE profile A1, which was also the most prevalent PFGE profile among the isolates analysed in this study as it was found in 37.9% of isolates.

Cluster B consisted of 30 isolates obtained from the samples collected almost exclusively at the subcontractors of FBO SI-C. Only two isolates within this cluster originated from subcontractors of FBO SI-A (Table 2). The most common PFGE profile in this cluster was B1, identified in 93.3% of the isolates. In addition, this PFGE profile was the second most prevalent in the present study, shared by 32.2% of the isolates.

Similar to cluster B, cluster C contained the isolates related to the subcontractors of a single FBO, in this case SI-A, with the exception of one isolate. The PFGE profile C1 was identified in 7 out of 10 isolates (Table 2).

The isolates connected to FBO SI-B grouped together in cluster D, mostly sharing the PFGE profile D1 (6/7 isolates). The smallest cluster E comprised three isolates from three farms related to FBO SI-A and SI-C (Table 2). Two unique PFGE profiles were identified, which occurred in 2010 on two farms (Figure 2).

Analysis of the isolates from holdings with multiple* S*. Infantis-positive samples revealed the following situation: in 8 out of 17 cases (at holdings SI-A-01, SI-A-04, SI-B-02, SI-C-01, SI-C-07, SI-C-09, SI-C-12, and SI-C-21; see Table 1), isolates with the same PFGE profile were retrieved from all samples collected within a respective holding, regardless of the time span between samplings (range 6 months to 4 years). Among these, a common profile (C1) has also been observed in different poultry houses of the same holding (SI-A-01) in the period of two years (Table 1). Isolates with distinct PFGE profiles were detected in nine cases, but it should be noted that different housing objects within the same holding were sampled in six cases (SI-A-02, SI-A-08, SI-A-13, SI-C-08, SI-C-22, and SI-X-01; see Table 1).

### 3.3. Biofilm Formation

**Figure 3:**
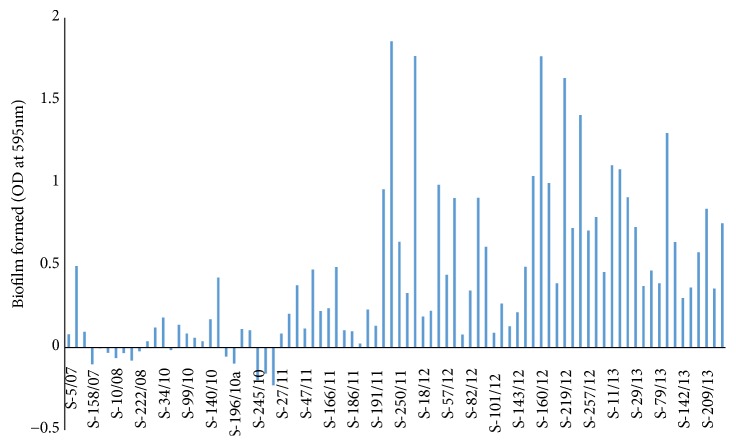
Average biofilm formation of 84* Salmonella* Infantis isolates from broilers, shown as optical density values measured at 595 nm (OD_595_).

Strains with OD_595_ values between 0 and 0.5 were considered as weak biofilm producers, strains with OD_595_ between 0.5 and 2.5 as medium to high biofilm producers, and strains with OD_595_ above 2.5 as very high biofilm producers. Average biofilm formation measured as OD_595_ for all strains tested was 0.42±0.17 (standard deviation). There were large differences between the strains regarding the ability to form biofilm (Figure 3).

**Figure 4:**
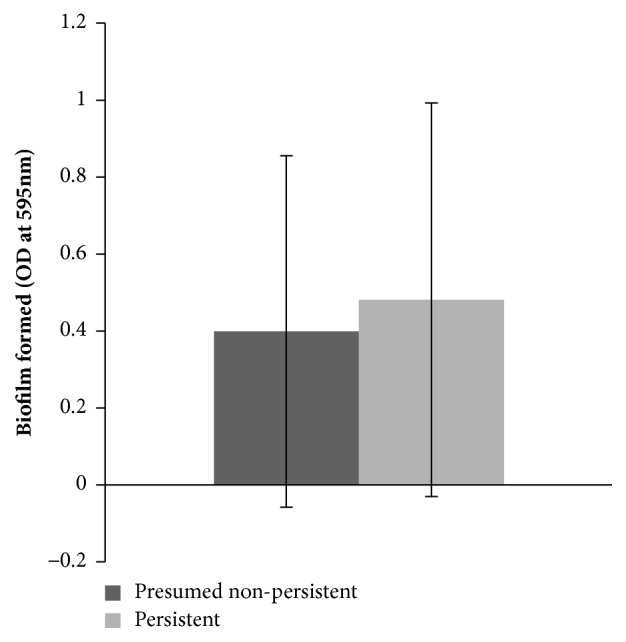
Biofilm formation by persistent* Salmonella* Infantis strains and presumably nonpersistent* Salmonella* Infantis strains. The results are shown as average optical density measured at 595 nm (OD_595_) from two independent experiments. The results are shown with standard deviation.

The average biofilm formation for persistent strains was higher than for presumably nonpersistent strains, but the difference was not significant as the standard deviation was large and the difference in ability to form biofilm was small (Student's* t*-test, p=0.44) (Figure 4).

**Figure 5:**
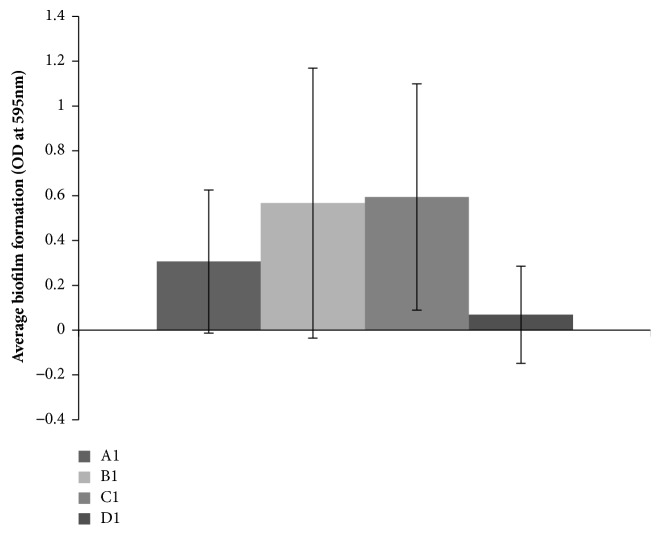
Biofilm formation by the different dominant PFGE profiles of* Salmonella* Infantis isolates, shown as optical density values at 595 nm (OD_595_). The results are given with the standard deviation of the strains within the PFGE profile.

Of the strains tested for biofilm formation (n=84), profiles A1 (n=32), B1 (n=28), C1 (n=7), and D1 (n=6) were the most dominant. The average biofilm formation between the profiles varied, but the standard deviation between the strains within one profile was large (Figure 5). Student's* t*-test analysis showed that profile B1 produced significantly more biofilm than profile A1 (p<0.05), and profile C1 produced significantly more biofilm than profile D1 (p<0.05). Due to the large standard deviation between the strains within one group, there was no significant difference between profiles A1 and C1 (p= 0.06), A1 and D1 (p= 0.09), B1 and C1 (p= 0.91), and B1 and D1 (p= 0.06).

Among the 84 strains included in biofilm formation testing, the two predominant resistance patterns were CipNxSSuT (n= 52, average OD_595_ = 0.50±0.19) and CipNxSuT (n=12, average OD_595_ = 0.33±0.15). There was no significant difference in biofilm formation between the strains with mentioned resistance patterns (Student's* t*-test, p=0.29).

## 4. Discussion

The number of* S*. Infantis-positive broiler flocks in Slovenia has steadily increased in the past eight years from 0.7% of positive flocks in 2010 to 11.5% in 2017 (Maja Bajt, personal communication). Detection of* S*. Infantis in minced meat, meat preparations, and meat products is not compliant with the EU regulation concerning the microbiological food safety. In the national zoonosis monitoring program from 2013 to 2015,* S*. Infantis was detected in 4.5% of the food samples of animal origin. The prevalence of* Salmonella* spp. was found to be the highest in fresh broiler meat and broiler meat preparations (28.4% and 26.7%, respectively), and the predominant* Salmonella* serovar was* S*. Infantis (92% and 100%, respectively) [16]. The increase of* Salmonella*-positive broiler flocks, which was due to increase of* S*. Infantis-positive flocks, raised the interest of veterinary authorities to conduct the study described herein, the first of its kind regarding* S. *Infantis in Slovenia.

On the EU level, as reported from all Member States in 2017,* S. *Infantis accounted for 46.5% and 50.6% of all* Salmonella* isolated from broiler flocks and broiler meat, respectively [2]. This indicates that this serovar has the capability to spread along the entire broiler production chain and can persist in the farm environment once it has become established.

The results from the present study demonstrated a high level of resistance to ciprofloxacin (76/87; 87.4%), sulfonamides (77/87; 88.5%), tetracyclines (77/87; 88.5%), nalidixic acid (76/87; 87.4%), and streptomycin (63/87; 72.4%). These findings are in concordance with the situation in Europe, as most EU Member States reported extremely high levels of resistance to ciprofloxacin and nalidixic acid (overall 94.1% and 94.1%, respectively). Overall resistance to sulfamethoxazole and tetracycline at the EU level was 78% and 75.6%, respectively [3]. In addition, >80% of* S*. Infantis isolates from broilers, tested for MDR at the EU level, were MDR and displayed a wide range of different MDR patterns. However, >97% MDR patterns included resistance to ciprofloxacin and/or nalidixic acid, as well as resistance to sulfamethoxazole and tetracycline. This was the most common resistance pattern in* S*. Infantis from broiler meat (72.7%) and broilers (78.4%) [3]. The results presented herein correlate with the data reported by EFSA as the vast majority of* S*. Infantis MDR patterns in Slovenia included resistance to the above-mentioned antimicrobials.

It seems that MDR in* S*. Infantis emerged in the new millennium. Gal-Mor et al. [17] reported notable differences between the strains isolated before and after 2007. Historical strains (before 2007) were either susceptible to all antimicrobials tested or resistant to only one antimicrobial agent. On the contrary, none of the more recent isolates (2007-2009) were susceptible to all antimicrobials tested and most of the isolates were MDR, which suggested resistance acquisition over time. Moreover, historical isolates did not share any obvious resistance pattern, while most of the more recent strains had a common combined resistance pattern. These findings correlate with the present study. Specifically,* S*. Infantis isolated in 2007 from the broilers raised at subcontractors of FBO SI-B were either susceptible to all antimicrobials tested or resistant to only one antimicrobial agent. Unfortunately, the number of isolates tested was limited, and for this reason it would be interesting to investigate in more detail the occurrence of antimicrobial resistance in* S*. Infantis isolates in Slovenia. Already in the following year, isolates with the most common MDR pattern (CipNxSSuT) emerged on the broiler farms and have been regularly detected in broiler flocks ever since, though occasionally complemented with the resistance to ampicillin. Interestingly, the isolates susceptible to all antimicrobials tested formed a small PFGE cluster D together with two isolates showing resistance to one antimicrobial each (Table 2). Otherwise, there was no correlation observed between the PFGE profiles and resistance patterns. The most prevalent PFGE profile A1 was associated with all MDR patterns, and the second most common profile B1 with the two most common MDR patterns (CipNxSSuT and CipNxSuT).

A high genetic homogeneity has been observed within the analysed* S. *Infantis isolates as 16 profiles were defined among 87 isolates, with a vast majority of them showing >90% profile similarity. These results are in correspondence with the findings from several other studies [18–20] including Nogrady et al. [21] who revealed close relatedness between the isolates (16 PFGE profiles in five clusters among 138 isolates); moreover, 66% of the isolates tested belonged to the same genetic clone, which emerged in Hungary in both humans and broilers. The genetic homogeneity of* S*. Infantis is supposed to be a consequence of possible clonal expansion and establishment of specific PFGE profiles [18, 19]. In a study by Hauser et al. [22] most isolates were assigned to two clusters, comprising 40.2% and 34.5% of isolates, respectively. These results are in accordance with the findings of the present study in which two PFGE profiles encompassed 37.9% and 32.2% of all isolates. The predominance could be explained either by the prevalence of a certain clone in the environment or by a common source of infection. Namely, the most frequently identified PFGE profile A1 was detected both at AOHs and at the subcontractors of FBOs SI-C and SI-A. The fact that the FBOs maintained their own breeding flocks ruled out the possibility of a common origin of animals reared by different FBOs. Therefore, the reason for a widespread dissemination of the PFGE profile A1 could be its prevalence in the environment, along with its temporal and genetic stability. Conversely, the case of PFGE profile B1, which was found almost exclusively (with one exception to the rule) at the holdings of SI-C subcontractors, pointed out a probable common source of infection (feed, water, and environment). In a previous study, conducted after the cleaning and disinfection of the broiler house before repopulation, we demonstrated the presence of* S*. Infantis in more than 50% of environmental samples taken, including floor, ventilators, drinking troughs, boots, and a high pressure washer (unpublished data). This indicates improper/ineffective sanitation procedures.

Biofilms increase microbial tolerance to chemical, physical, and biological agents, and the ability to form biofilms has been identified as an important but not the only contributing factor for the persistence of bacteria in the environment [10]. Being a good biofilm producer only gives an advantage. Several hypotheses are related to the high persistence of* Salmonella* in poultry houses, such as the absence of standardised cleaning and disinfection guidelines, inaccurate use of disinfectants, incorrect hardness and temperature of cleaning water, high contents of protective compounds in poultry houses, and biofilm development, as reviewed by Marin et al. [8]. However, Marin et al. [8] demonstrated that the disinfectants used in infected poultry houses are more important than the capacity of* Salmonella* to form biofilm.

Biofilm-forming ability of* Salmonella* may vary both between and within serovars. Certain serotypes, e.g.,* S*. Agona, showed significantly more biofilm formation in comparison to other serotypes [10, 11]. The method for testing biofilm-forming capacity in the present study was based on a previously described method with a modification of the percentage of overnight culture added to the biofilm growth medium. In the present assay, a 10% overnight culture (20 *μ*l + 180 *μ*l) was used, while in the study by Vestby et al. [10], as much as 23% (30 *μ*l + 100 *μ*l) was used. This has been seen to have very little influence on the final amount of biofilm formed after two days of incubation. The average biofilm produced by three* E. coli* strains and four* Salmonella* strains after two days when a 23% overnight culture was added was 1.57 (OD_595_). When adding as little as a 0.1% overnight culture of the same strains, the OD_595_ was 1.78 (Student's* t*-test, p=0.54) (unpublished data). For this reason, it is considered as likely that adding a 10% instead of a 23% overnight culture has very limited impact on the result.

*S*. Infantis from chicken carcasses was in general classified as a moderate biofilm producer but showed high variability in the biofilm-forming ability [10], which is similar to* S*. Infantis isolates of pork origin [23]. The overall biofilm-forming ability of the isolates collected in the present study was determined as weak/moderate (median OD_595_ 0.28). The majority of isolates (73.8%) were weak biofilm producers, and none of the isolates exhibited a very high biofilm-forming ability. There were also substantial differences noted in the ability of tested isolates to produce biofilm (OD_595_ range from -0.229 to 1.857). We also attempted to compare the biofilm-forming ability of the persistent and presumably nonpersistent strains. Even though average biofilm formation for persistent strains was higher than for presumed nonpersistent strains, the difference was not significant as the standard deviation was large.

Four holdings were presumed to have persistent* S*. Infantis strains as the same PFGE profile was repeatedly identified in strains obtained in different time periods (range 1 year 7 months to 4 years 1 month) in the same broiler house. The presumably persistent strains were of PFGE profiles A1, B1, and C1, and of resistance patterns CipNxSSuT and CipNxSuT. There was a variation observed regarding the biofilm-forming ability of the isolates collected within individual holdings with presumably persistent strains. It seems that the biofilm-forming ability increased with time, as the isolates from 2012 and 2013 showed higher OD_595_ values compared to the isolates before 2010. There was no obvious reason found for this observation except the differences in storage and number of passages. However, Schoneville et al. [12] reported that 10 passages of selected isolates did not affect their phenotypic biofilm-forming capacity.

## 5. Conclusions

The results of this study complement the current knowledge on* S*. Infantis in broiler flocks. The strains isolated after 2007 were characterised by a high resistance to antimicrobials showing a widespread MDR pattern, genetic homogeneity, and low average biofilm-forming ability. It seems that, as reported previously for* S*. Infantis from Hungary which was characterised as a poor biofilm producer despite being the single most dominant serotype in the country [12],* S*. Infantis persistence on broiler farms could be more related to its widespread occurrence in broiler production and ineffective disinfection protocols than to its ability to form biofilm. Biosafety guidelines and disinfection practices on the farms should therefore be reviewed, and radical measures to limit the spread of the microbe should be discussed.

## Figures and Tables

**Table 1:** Overview of PFGE profiles, resistance patterns, and biofilm formation of *Salmonella* Infantis isolates from broiler farms collected between 2007 and 2013.

**No**	**Farm ID** ^**a**^	**Isolate ID**	**Sampling time**	**Location**	**PFGE profile**	**Resistance pattern** ^**b**^	**ABF** ^**c**^
1	SI-A-01	S-163/11	Sept 2011	broiler house 1	C1	CipNxSSuT	0.221
2		S-149/12	Aug 2012	broiler house 6	C1	CipNxSSuT	1.04
3		S-11/13	Feb 2013	broiler house 5	C1	CipNxSSuT	1.105
4		*S-209/13* ^d^	Sept 2013	broiler house 4	C1	CipNxSSuT	0.842

5	SI-A-02	S-99/10	May 2010	broiler house 1	B3	CipNxSuT	0.0855
6		S-243/10	Sept 2010	broiler house 2	A1	CipNxSSuT	0.113

7	SI-A-03	S-205/11	Nov 2011	na^e^	C1	CipNxSSuT	0.959

8	SI-A-04	S-257/12	Nov 2012	broiler house 1	A1	CipNxSSuT	0.7095
9		S-79/13	May 2013	broiler house 1	A1	CipNxSSuT	0.3895

10	SI-A-05	S-141/12	Aug 2012	na	A2	ACipNxSSuT	0.129

11	SI-A-06	S-78/13	May 2013	na	A1	CipNxSSuT	0.4665

12	SI-A-07	S-244/10	Nov 2010	na	A1	CipNxSSuT	0.1055

13	SI-A-08	S-196/10a	Oct 2010	broiler house 2	C1	CipNxSuT	-0.0975
14		S-245/10	Nov 2010	broiler house 1	B1	sensitive	-0.1975
15		S-83/12	May 2012	broiler house 1	C3	CipNxSSuT	0.908

16	SI-A-09	S-101/12	June 2012	na	C1	CipNxSSuT	0.089

17	SI-A-10	S-72/11	June 2011	na	A1	CipNxSSuT	nd^f^

18	SI-A-11	S-160/13	July 2013	na	A1	CipNxSSuT	0.3645

19	SI-A-12	S-74/13	May 2013	na	A1	ACipNxSSuT	0.373

20	SI-A-13	S-140/10	July 2010	broiler house 1	A1	CipNxSSuT	0.1715
21		S-261/12	Dec 2012	broiler house 2	A3	CipNxSuT	0.7915

22	SI-A-14	S-251/12	Nov 2012	na	C2	CipNxSuT	1.4095

23	SI-A-15	S-142/13	June 2013	na	A1	CipNxSSuT	0.3

24	SI-A-16	S-210/13	Sept 2013	na	E2	A	0.358

25	SI-A-17	S-220/12	Nov 2012	na	A1	CipNxSSuT	0.724

26	SI-B-01	S-101/08	Apr 2013	na	D1	A	-0.033

27	SI-B-02	S-5/07	nr^g^	nr	D1	S	0.081
28		S-12/07	nr	nr	D1	sensitive	0.4945

29	SI-B-NN	S-131/07	nr	nr	D2	sensitive	0.096
30		S-158/07	nr	nr	D1	sensitive	-0.1015
31		S-271/07	nr	nr	D1	sensitive	-0.005
32		S-222/08	Sept 2008	broiler house 1	D1	sensitive	-0.023

33	SI-C-01	S-27/11	Mar 2011	broiler house 1	B1	CipNxSuT	0.0855
34		S-185/11	Oct 2011	broiler house 1	B1	CipNxSSuT	0.105
35		S-250/11	Dec 2011	broiler house 1	B1	CipNxSSuT	0.6415
36		S-18/12	Jan 2012	broiler house 1	B1	CipNxSSuT	0.1885
37		S-53/12	Mar 2012	broiler house 1	B1	CipNxSSuT	0.9875
38		S-160/12	Sept 2012	broiler house 1	B1	CipNxSSuT	1.7655
39		*S-219/12* ^d^	Nov 2012	broiler house 1	B1	CipNxSSuT	1.6335

40	SI-C-02	S-184/11	Oct 2011	na	B1	CipNxSuT	0.4885

41	SI-C-03	S-129/10	June 2010	broiler house 1	B1	CipNxSSuT	0.0375
42		S-124/13	June 2013	broiler house 1	E1	sensitive	1.3005

43	SI-C-04	S-186/11	Oct 2011	na	B1	CipNxSSuT	0.099

44	SI-C-05	S-187/13	Aug 2013	na	A1	CipNxSSuT	0.578

45	SI-C-06	S-12/12	Jan 2012	na	A1	CipNxSSuT	0.33

46	SI-C-07	S-188/11	Oct 2011	broiler house 1	B1	CipNxSSuT	0.02375
47		S-87/12	May 2012	broiler house 1	B1	CipNxSSuT	0.6115
48		S-274/12	Dec 2012	broiler house 1	B1	CipNxSSuT	0.2845

49	SI-C-08	S-207/11	Nov 2011	broiler house 2	B1	CipNxSSuT	1.857
50		S-180/12	Oct 2012	broiler house 1	A1	CipNxSSuT	0.389

51	SI-C-09	S-204/08	Aug 2008	broiler house 1	A1	CipNxSSuT	-0.079
52		S-97/10	Apr 2010	broiler house 1	A1	CipNxSuT	0.1375
53		S-40/11	Apr 2011	broiler house 1	A1	CipNxSuT	0.377
54		*S-175/12* ^d^	Oct 2012	broiler house 1	A1	CipNxSSuT	0.997

55	SI-C-10	S-189/11	Oct 2011	na	B1	CipNxSSuT	0.23

56	SI-C-11	S-47/11	May 2011	na	A1	CipNxSSuT	0.116

57	SI-C-12	S-82/12	May 2012	broiler house 1	A1	CipNxSSuT	0.3465
58		S-21/13	Feb 2013	broiler house 1	A1	CipNxSSuT	0.9105

59	SI-C-13	S-41/12	Mar 2012	na	A1	CipNxSSuT	0.223

60	SI-C-14	S-30/11	Apr 2011	broiler house 1	A1	CipNxSuT	0.2055
61		S-14/12	Jan 2012	broiler house 1	B1	CipNxSSuT	1.767

62	SI-C-15	S-3/08	Dec 2017	nr	A1	CipNxSSuT	-0.031
63		S-10/08	nr	nr	A1	CipNxSSuT	-0.063
64		S-213/09	Aug 2009	nr	A1	CipNxSSuT	0.0385
65		S-1/10	Dec 2009	nr	G	CipNxSuT	nd
66		S-59/10	Mar 2010	broiler house 1	A1	CipNxSuT	-0.0155
67		S-48/11	May 2011	broiler house 1	A1	CipNxSuT	0.4735
68		S-191/11	Nov 2011	broiler house 1	C4	CipNxSSuT	0.132
69		S-111/12	June 2012	broiler house 1	A1	CipNxSSuT	0.2665

70	SI-C-16	S-57/12	Apr 2012	na	B1	CipNxSSuT	0.4405

71	SI-C-17	S-26/11	Mar 2011	na	A1	CipNxSSuT	-0.229

72	SI-C-18	S-143/12	Aug 2012	na	B1	CipNxSSuT	0.2135

73	SI-C-19	S-149/10	Aug 2010	na	E2	CipNxSSuT	0.425

74	SI-C-20	S-20/13	Feb 2013	na	A1	SSuT	1.081

75	SI-C-21	S-223/09	Aug 2009	nr	B1	CipNxSuT	0.122
76		S-119/10	June 2010	broiler house 1	B1	CipNxSuT	0.06
77		S-7/11	Feb 2011	broiler house 1	B1	CipNxSSuT	-0.158
78		S-166/11	Sept 2011	broiler house 1	B1	CipNxSSuT	0.2375
79		S-60/12	Apr 2012	broiler house 1	B1	CipNxSSuT	0.9065
80		S-144/12	Aug 2012	broiler house 1	B1	CipNxSSuT	0.4895
81		S-29/13	Mar 2013	broiler house 1	B1	CipNxSSuT	0.731
82		*S-215/13* ^d^	Sept 2013	broiler house 1	B1	CipNxSSuT	0.754

83	SI-C-22	S-251/11	Dec 2011	broiler house 1	B2	CipNxSuT	nd
84		S-138/13	June 2013	broiler house 2	B1	CipNxSSuT	0.6405

85	SI-X-01	S-34/10	Feb 2010	broiler house 1	F	CipNxSSuT	0.1815
86		S-176/10	Sept 2010	broiler house 2	A1	CipNxSSuT	-0.0545

87	SI-X-02	S-73/12	Apr 2012	na	A1	ACipNxSSuT	0.079

^a^The origin of the isolates is indicated by farm ID, consisting of capital letters SI-A, SI-B, SI-C (standing for three major food business operators, FBOs), and SI-X (standing for autonomously operating holdings, AOHs); while digits designate individual subcontractors of a certain FBO.  ^b^Resistance pattern code: S, streptomycin, Su, sulfamethoxazole, Cip, ciprofloxacin, Nx, nalidixic acid, A, ampicillin, and T, tetracycline.  ^c^Average biofilm formation measured as optical density at 595 nm (OD_595_).  ^d^Isolates classified as persistent.  ^e^Not applicable.  ^f^Test not performed.  ^g^Data not recorded.

**Table 2:** Occurrence of *Salmonella* Infantis PFGE profiles at broiler chicken holdings from 2007 to 2013.

PFGE profile	No. of isolates (n=87)	FBO/AOH^a^	Years
A1	33	SI-A (n^b^ =11), SI-C (n=12), SI-X01 (n=1), SI-X02 (n=1)	2008-2013
A2	1	SI-A	2012
A3	1	SI-A	2012

B1	28	SI-A (n=1), SI-C (n=12)	2009-2013
B2	1	SI-C	2011
B3	1	SI-A	2010

C1	7	SI-A	2010-2013
C2	1	SI-A	2012
C3	1	SI-A	2012
C4	1	SI-C	2011

D1	6	SI-B	2007-2008
D2	1	SI-B	2007

E1	1	SI-C	2013
E2	2	SI-A, SI-C	2013, 2010

F	1	SI-X-01	2010

G	1	SI-C	2010

^a^Food business operator/autonomously operating holding.  ^b^Number of subcontracting farms.

## Data Availability

The data used to support the findings of this study are available from the corresponding author upon request.
